# Bilateral Section of Cervix Uteri

**Published:** 1875-10

**Authors:** Thad. A. Reamy


BILATERAL SECTION OF CERVIX UTERI.—*By Thad. A. Reamy, M.D.* *Results.*—*Cured* one—a virgin, after four months subsequent treatment. She married four years after operation, and became a mother. Has borne two children. Labors easy and natural. Was attended during first confinement by myself. No cicatricial deformity of os.

Relieved for periods varying from three to six months after operation—4. In each of these cases the symptoms returning with more or less violence. All benefits from the operation lost. And in two of these four cases there was subsequently eversion of the anterior and posterior lips with ulceration dependent upon the division, reaching a condition more imperatively demanding opertive procedures for relief than the original difficulty.

Permanently relieved but not cured—3.

Followed by pregnancy going to term—1.

Followed by pregnancy and aborting apparently from condition of cervix—1.

No improvement whatever—3.

Operation followed by peri-uterine and pelvic cellulitis, imperilling the patients lives—2 In one of these cases the uterus becoming immobile finally, in a retroverted position.

These cases were under my observation directly and indirectly for periods varying from six months to several years. Two of these patients have since died of other diseases: and possibly others. Several of these patients have now of course passed from my observation, and their subsequent history might possibly change to some extent my final conclusions as to the results of the treatment employed.

If it is the pleasure of the Academy I will in the near future give the results of other plans of treatment expressed by a large number of cases. Results which have, to me at least, been far more satisfactory. In conclusion, I have only to say, I consider bilateral section of the uterine cervix unwarranted, except in rare cases of cervical deformity attended with anteflexion.* Unwarranted, first, because it does but little good, and in a majority of cases no good whatever. 2nd. Because it often induces conditions more to be deplored than those for which it is employed—eversion, etc.

---

* Of course incisions for relief of uterine fibroids are excluded from this denial of propriety of the practice; these incisions I make, but I do not confine them to the cervical lateral line.

3d. Because, except under the most favorable auspices, the operation is attended with danger to life in its consequences. 4th. Because other means far safer and far more efficient are at our command.—*Clinic.*

